# Adaptive aggregation by spider mites under predation risk

**DOI:** 10.1038/s41598-017-10819-8

**Published:** 2017-09-06

**Authors:** Lena Dittmann, Peter Schausberger

**Affiliations:** 1 Group of Arthropod Ecology and Behavior, Department of Crop Sciences, University of Natural Resources and Life Sciences, Gregor-Mendel-Straße 33, 1180 Vienna, Austria; 2 Department of Behavioural Biology, University of Vienna, Althanstraße 14, 1090 Vienna, Austria

## Abstract

Grouping together is a commonly observed anti-predator strategy. Possible anti-predator benefits of aggregation include the encounter/avoidance effect for visually hunting predators and the dilution effect, together dubbed attack abatement. Possible costs opposing the dilution effect are easier detection of aggregated than scattered individuals. The benefits of attack abatement, and opposing costs after group detection, are poorly understood for chemosensory predator-prey interactions. We tackled this issue by assessing the aggregation behavior of spider mites *Tetranychus urticae* under predation risk emanating from predatory mites *Phytoseiulus persimilis*. We examined whether adult spider mite females aggregate more tightly when perceiving predator cues (traces left and eggs), representing graded risk levels, and whether grouping enhances survival in physical predator presence. The spider mites aggregated more tightly and were more active in presence than absence of predator cues. Grouped spider mites were less likely and later detected and attacked than scattered spider mites. Moreover, encounter and attack of one group member did not increase the risk of other members to be attacked, as compared to scattered spider mites. To the best of our knowledge, our study is the first rigorous documentation of the adaptive benefit of tightened prey aggregation towards a purely chemosensorily hunting predator.

## Introduction

Predation is a strong selective force shaping prey morphology, life history and/or behavior. Prey commonly evolve specific behaviors to avoid or minimize predation risk^1^, which are accordingly dubbed anti-predator behaviors. Anti-predator behaviors comprise all strategies of prey aiming at preventing detection by predators or avoiding predation after detection. Across animal taxa, anti-predator strategies range from habitat selection over increased vigilance for an early predator detection to active defense mechanisms confusing or repelling predators^1^. One crucial aspect of any predator-prey interaction and thus evolution of anti-predator strategies is whether animals live solitary or in groups^2–4^.

Enhanced anti-predator functions are considered important drivers of group-formation and -living^2–4^. Anti-predator benefits of group-living may arise from various, mutually non-exclusive, phenomena such as cooperative defense^5^, increased vigilance^6^, possibly combined with alarm signals^7, 8^, predator confusion^9^, the dilution effect^10, 11^ and/or reduced encounter likelihood (dubbed the encounter or avoidance effect)^4, 11^. The dilution effect assumes reduced predation risk of individual group members, due to presence of alternative targets, upon attack of a predator that cannot capture all group members^10, 11^. The encounter or avoidance effect applies primarily to visually hunting predators and assumes that a group, although being more conspicuous, is often, but not necessarily always^4, 12, 13^, less likely detected than the same number of scattered individuals in the same area^4, 11, 14^. Regarding the encounter/avoidance effect, Ruxton & Johnsen^15^ used a modeling approach to show that the likelihood of encounter/detection by visually hunting predators increases disproportionally to the number of prey group members, i.e. at a lower rate than prey group size. Pertinent experimental examples come from visually hunting sticklebacks, which detect aggregated prey less likely than the same number of prey scattered in the same area^16^, and detection rate increasing disproportionally to increasing group size^17^. Turner & Pitcher^11^ postulated that only the combined effect of encounter/avoidance and dilution - dubbed attack abatement - reduces the predation risk of individual group members. Attack abatement assumes that the attack rate of individual group members increases at a slower rate upon predator encounter than the predator encounter rate increases with group size. In any case, possible benefit-cost trade-offs arising from the encounter/avoidance effect, the dilution effect or attack abatement inherently depend on the predators’ prey searching and capturing strategies.

Predators can be visual, mechano- and/or chemosensory oriented hunters^18–21^ and their sensory modalities and search strategies (co-)shape the anti-predator strategies of prey. For example, prey of visually hunting predators typically benefit from crypsis and hiding, whereas prey of predators using mechanosensory, such as auditory, cues, benefit from reduced activity. For all of them, grouping together or tightening the aggregation level are possible additional or alternative primary anti-predator strategies^4^. However, most theoretical and experimental work on group-specific anti-predator functions such as attack abatement focused on predator-prey interactions mediated by visual cues. Whether the encounter/avoidance effect and attack abatement hold true for purely chemosensorily hunting predators is poorly understood^4^. Treisman^14^ suggested that olfactorily hunting predators should detect a group of prey individuals from a proportionally greater distance (proportional to the number of group members), relative to a single prey individual. If so, the encounter/avoidance effect would not necessarily hold true for purely chemosensorily based predator-prey interactions. In general, the scaling rules for olfactory cues are much more difficult to determine than those for visual cues, due to the complexity arising from turbulence, velocity and convection^22^. While Kunin^23^ suggested that attraction of herbivores from the distance should be proportional to the number of odor sources and thus group size, Andersson *et al*.^22^ determined that the distance at which moths can find an odor source increases proportional to the square root of the number of odor sources, but not proportional to the actual number.

Here, we assessed whether modifying the aggregation level is an adaptive anti-predator response of the group-living two-spotted spider mite *Tetranychus urticae* to cues of its most important predator *Phytoseiulus persimilis* ^24–26^. Tightened aggregation is a typical form of anti-predator behavior observed in many group-living animals^27–33^. For *T. urticae*, Yano^34^ suggested that more tightly aggregated spider mites benefit from denser webbing, but the anti-predator function of aggregation itself, without the webbing function, has not yet been scrutinized. *T. urticae* is a globally distributed, extremely generalist herbivore with >1000 different host plant species^35^. Spider mites are group-living and form patchily distributed colonies, consisting of a few to thousands individuals, on their host plants. They possess spinning glands in their pedipalps and constantly spin threads while walking^36^. The webs are highly important for protection against predators, for regulating local micro-climatic conditions on the leaf surface, and for protection against wind, rain, dust and other environmental hazards^34, 37–39^. To enhance group formation, *T. urticae* follow the threads of conspecifics; group members benefit from Allee effects^34, 39–41^.

The specialist predatory mite *P. persimilis* feeds exclusively on tetranychid spider mites and copes well with the dense webs produced by *T. urticae* ^42, 43^. *P. persimilis* is eyeless and predominantly uses volatile and/or tactile chemosensory cues for orientation, locating and recognizing its prey^44, 45^. While *T. urticae* is relatively defenseless against *P. persimilis*, once detected and encountered, the spider mites evolved a suite of primary anti-predator behaviors to reduce the chance of detection. Most importantly, *T. urticae* is able to recognize the past and immediate presence of phytoseiid predators including *P. persimilis* through chemical traces such as metabolic waste products or footprints left by the predators on the plant surface^46^. The spider mites avoid plants or plant parts occupied by *P. persimilis* ^47, 48^ and are able to assess the relative risk of predation when exposed to cues of low to medium risk generalist and high risk specialist predatory mite species^26, 49^. In general, anti-predator behaviors can be fixed, and thus performed in a similar way under every circumstance, or can be risk-dependent^50–52^. Apart from the benefits, which are mostly enhanced survival of the prey individuals themselves or their progeny, every anti-predator behavior also incurs costs. Energy and time is spent for increased activity, enhanced vigilance and/or defense, which could otherwise be used for other life activities such as foraging or reproduction^1, 53, 54^. Therefore, due to the inherent costs of every anti-predator behavior, prey are expected to be able evaluating the level of predation risk and adjust their behavior accordingly.

The first objective of our study was determining if adult *T. urticae* females respond to predation risk posed by cues of *P. persimilis* with tighter aggregation and, if so, if the aggregative response varies with the level of predation risk. The second objective was verifying if an aggregated distribution indeed enhances the survival chances of the spider mites under immediate high predation risk posed by a hungry *P. persimilis* female, as compared to scattered distribution. To this end, we conducted two experiments to investigate (1) the aggregation level of *T. urticae* in response to various combinations of traces and eggs of *P. persimilis*, and (2) the survival chances of aggregated versus scattered *T. urticae* females in physical presence of a *P. persimilis* female.

## Materials and Methods

### Rearing

Two-spotted spider mites *T. urticae* were reared on whole common bean plants, *Phaseolus vulgaris*, under room conditions (23 ± 2 °C, 60 ± 10% relative humidity and 16:8 h L:D). Bean plants were grown in a substrate mixture containing 75% soil and 25% expanded clay in a walk-in environmental chamber (25 ± 2 °C, 60 ± 10% relative humidity, and a photoperiod of 16:8 h L:D). Infested plants were kept in a separate room from the clean plants used for the experiments. Detached un-infested trifoliate leaflets were used in the experiments.

The laboratory population of the predatory mite *P. persimilis* was originally founded by specimens collected on the coast of Oregon, USA, and reared on a detached bean leaf arena infested by *T. urticae*. The detached leaf arena consisted of a primary leaf of *P. vulgaris* placed upside down onto filter paper covering a water-saturated foam cube (15 × 15 × 4 cm), placed inside a plastic box (20 × 20 × 5 cm) half-filled with tap water. The edges of the leaf were covered with moist tissue paper confining the mites to the arena. The plastic box was placed into a larger plastic tray (45 × 34 × 9 cm), half-filled with tap water containing a drop of dishwashing detergent to reduce the surface tension. Every second to third day the predatory mites were provided with spider mites brushed off infested leaves onto the rearing arena.

### Pre-experimental treatment

In both experiments, we used standardized circular bean leaf discs (Ø 2.2 cm) as experimental arenas. Leaf discs were punched out of trifoliate leaflets such that every disc had a vein in the middle. Each leaf disc was placed upside down onto a water-saturated foam cuboid (2.0 × 2.0 × 1.5 cm) inside a cylindrical compartment (Ø 3.5 cm, 2.0 cm high) of a plastic cartridge (12.5 × 8.0 × 2.0 cm), each containing six separate compartments. Foam cuboids were fixed at the bottom of the compartments using vaseline and the compartments filled with tap water to the upper edge of the cuboids. Thereby, each leaf disc was completely surrounded by water creating a barrier for the mites.

### Aggregation under predation risk (experiment 1)

In the first experiment, we assessed the aggregation level of adult spider mite females and their eggs in dependence of the level of predation risk (no, low, medium, high) posed by *P. persimilis*. Graded predation risk levels were created by absence/presence of *P. persimilis* cues, such as metabolic waste products, chemical footprints and/or eggs, left on the leaf surface by the predators.

Before the experiment, each leaf disc either received one well-fed gravid female of *P. persimilis*, transferred from the rearing by using a fine moistened brush, or was left blank. The predator females were kept on the discs without prey for 15 h to leave traces such as metabolic waste products and possibly chemical footprints, and eggs, all of which are cues indicating predation risk for the spider mites^24, 26, 48^. We prepared four different types of leaf discs, differing in the level of predation risk: (1) discs with predator traces and five predator eggs, representing high predation risk, (2) discs with predator traces and two predator eggs, representing medium risk, (3) discs with predator traces but without eggs, representing low risk, and (4) discs without any predator cues, representing no risk (control). For treatment (1), some predator eggs from the predator rearing were manually added onto discs because the females were not able to produce five eggs during 15 h.

To start the experiment, five adult spider mite females, randomly withdrawn from the rearing, were placed onto each leaf disc. Subsequently, we observed and recorded the position and activity (moving/stationary) of the spider mite females every 30 min for 3.5 h in total, and then again after 24 h. At each observation, the position of the mites was marked on paper sketches of the leaf discs and their inter-individual distances measured after the experiment. After 3.5 and 24 h, we additionally recorded the number and position of eggs laid by the spider mite females. Spider mite females that had left the leaf disc during the initial 3.5 h of the experiment, which was, on average, less than 1 individual per disc across treatments, were replaced or, if still alive, rescued from the water and returned onto the disc. Leaf discs harboring only one or no spider mite female after 24 h were discarded. Each treatment was replicated 26 to 28 times; each leaf disc, each spider mite female and each predator female was used only once.

### Survival in dependence of aggregation (experiment 2)

In the second experiment, we compared the detectability and survival of four grouped versus four scattered spider mite females, fixed on the surface of a leaf disc, in presence of a gravid predatory mite female *P. persimilis*. The spider mite females were randomly withdrawn from the rearing, and fixed on the surface of the discs by placing them on top of individual vaseline drops, applied by a dissection needle. Fixation did not kill the spider mites. The vaseline drops were completely covered by the fixed spider mites and did not hinder the predatory mites in approaching and touching the spider mites. The “scattered” treatment consisted of four adult spider mite females placed solitarily at equidistance on a disc, with one female placed in the center of each quarter, halfway between the edge and the center of the disc. The “grouped” treatment consisted of four adult spider mite females placed in a group, at mutual distances of 1 mm, in one quarter of the leaf disc, halfway between the edge and the center of the disc.

Before the experiment, gravid females of *P. persimilis*, to be used in the experiment, were singly placed for 15 to 18 h into empty acrylic cages for starvation. Each cage consisted of a cylindrical chamber (Ø 1.5 cm, 0.3 cm high) drilled into an acrylic plate (8.0 × 3.5 × 0.3 cm). The chambers were closed by gauze on the bottom side and by a microscope slide fixed with rubber bands on the upper side^55^. To prevent dehydration of the mites, the acrylic cages were placed on a grid above tap water inside a plastic box (25.0 × 16.5 × 5.0 cm) stored in a climate chamber at 20 ± 1 °C, 60 ± 5% relative humidity and 16:8 h L:D. To start the experiment, one starved gravid *P. persimilis* female was placed onto each leaf disc harboring either four scattered or four grouped spider mite females, using a fine moistened brush. After releasing the predator female, the time of first encounter with a spider mite, the time of first attack on a spider mite and the predator activity was recorded every 10 min during the 1st h and every 20 min during the 2nd and 3rd h, for 3 h in total. First encounter was scored when the predator touched a spider mite with the first pair of legs; first attack was scored when the predator successfully grasped a spider mite and started to suck it out. Each treatment, “grouped” and “scattered”, was replicated 24 times; each leaf disc, each spider mite female and each predator female was used only once.

### Statistical analyses

SPSS® Version 23 (IBM Corp., USA) was used to analyze the results of both experiments.

In experiment 1, we used generalized estimating equations (GEE; normal distribution, identity link) to compare the mean inter-individual distances among the four spider mite females of a disc (one value per disc and time) among the four treatments over time (used as auto-correlated inner subject variable). Before analysis, the inter-individual distances were adjusted by a correction factor accounting for the number of spider mite females, out of four, present on the leaf disc at each point of observation. The correction factors were calculated by fractionating the total leaf disc area (radius of 11 mm) into non-overlapping virtual areas available to each individual present on the disc. Assuming a virtual circle around each individual, the radii of the individual circles were related to each other in dependence of the number of individuals on the disc. Accordingly, the correction factor was 1 for five, 0.89 for four, 0.77 for three, and 0.62 for two present females. Egg numbers (Poisson distribution, log link) and egg aggregation (using an egg dispersion index; binomial distribution, counts of events, log link) after 3.5 and 24 h were analyzed by separate generalized linear models (GLMs). The egg dispersion index was calculated by dividing each disc into a 16-sectored raster and counting the number of sectors harboring at least one egg. Thus, the lower the index the tighter egg aggregation.

In experiment 2, we used Cox hazard regressions to compare the likelihood and time elapsed until first encounter and first attack between the “scattered” and “grouped” treatments. Separate GLMs (Poisson distribution, log link) were used to analyze the number of killed spider mites during the 3 h experimental period, including and excluding discs without any killed spider mite.

### Data availability

The datasets generated during and/or analyzed during the current study are available from the corresponding author on reasonable request.

## Results

### Aggregation under predation risk (experiment 1)

**Figure 1:**
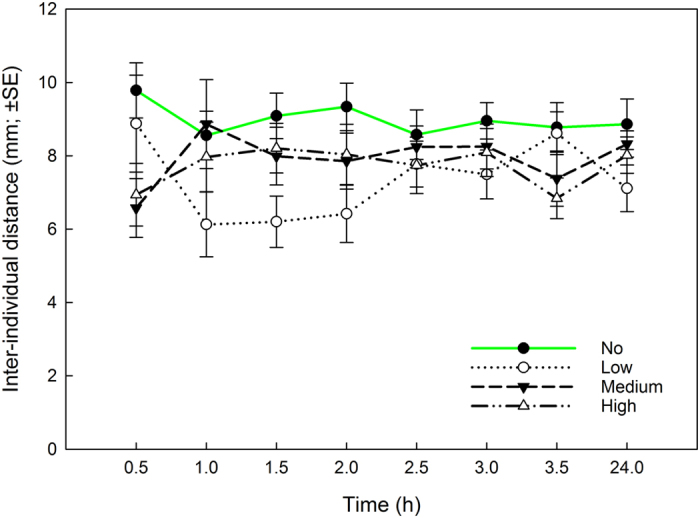
Inter-individual prey distances in dependence of predation risk. Inter-individual distances among five spider mite females on leaf discs presenting graded predation risk levels (no, low, medium, high) posed by cues of the predatory mite *Phytoseiulus persimilis*.

The inter-individual distances of the spider mites varied significantly with presence/absence of predatory mite cues on the leaf discs (Fig. 1; GEE: *Wald ӽ*_3_ *²* = 17.368, *P* = 0.001) but remained constant across time and treatments (treatment nested in time; *Wald ӽ*_28_ *²* = 37.406, *P* = 0.11). Post-hoc least significant difference (LSD) tests revealed that the spider mites aggregated more tightly in presence than absence of predatory mite cues (no versus low, medium, or high; *P* < 0.01 for every pairwise comparison). The distances were similar among treatments with predatory mite cues (low versus medium versus high predation risk; *P* > 0.21 for every pairwise comparison).

**Figure 2:**
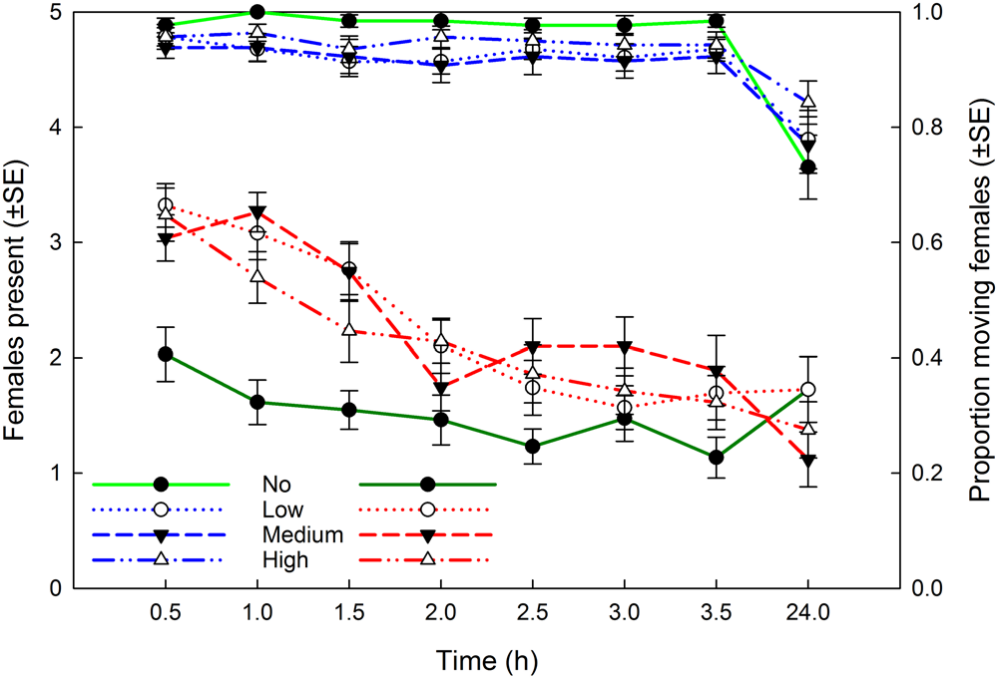
Prey presence and activity in dependence of predation risk. Presence (blue lines with light green for the control treatment) and activity (red lines with dark green for the control treatment) of five spider mite females on leaf discs presenting graded predation risk levels (no, low, medium, high) posed by cues of the predatory mite *Phytoseiulus persimilis*.

Albeit slightly lower in treatments with predatory mite cues, the number of spider mite females present on the leaf disc was not significantly affected by presence/absence of predatory mite cues (Fig. 2; GEE: *Wald ӽ*_3_ *²* = 5.084, *P* = 0.17) but varied significantly over time (treatment nested in time; *Wald ӽ*_3_ *²* = 88.359, *P* < 0.001). In all four treatments, almost all five females were present on the disc until 3.5 h, whereas, on average, one to two females had left the disc after 24 h. Activity of the spider mite females was affected by the presence of predatory mite cues (Fig. 2; GEE: *Wald ӽ*_3_ *²* = 39.02, *P* < 0.001) and developed significantly differently among treatments over time (treatment nested in time; *Wald ӽ*_28_ *²* = 387.12, *P* < 0.001). The spider mite females were more active in presence than absence of *P. persimilis* cues, no matter of the type of cues. Pairwise comparisons revealed that the control treatment (no risk) differed from all predatory mite cue treatments (*P* < 0.001), whereas the three predatory mite cue treatments (low, medium, and high risk) did not differ among each other (*P* > 0.22 for each pairwise comparison). Except after 24 h, the females were much more active in treatments with predatory mite cues than in the control treatment. After 1 h (*P. persimilis* traces and *P. persimilis* traces plus 5 eggs) and 1.5 h (*P. persimilis* traces plus 2 eggs), respectively, the spider mites began to gradually reduce their activity (Fig. 2).

**Figure 3:**
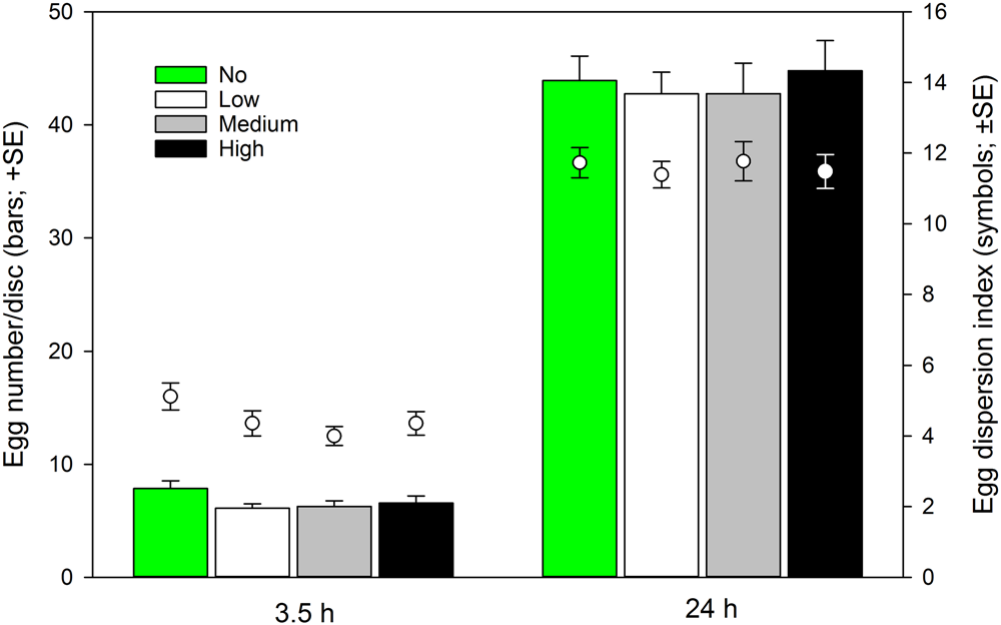
Prey egg production and aggregation in dependence of predation risk. Number and aggregation of eggs laid by five spider mite females within 3.5 and 24 h on leaf discs presenting graded predation risk levels (no, low, medium, high) posed by cues of the predatory mite *Phytoseiulus persimilis*. The egg dispersion index represents the number of virtual leaf disc sectors, out of 16, harboring at least one spider mite egg.

The number of eggs produced by the spider mite females was marginally significantly lower in treatments with than without predatory mite cues after 3.5 h (Fig. 3; GLM: *Wald ӽ*_3_ *²* = 7.339, *P* = 0.06) but was similar across treatments after 24 h (*Wald ӽ*_3_ *²* = 1.839, *P* = 0.61). Egg aggregation was marginally significantly tighter in treatments with than without predatory mite cues after 3.5 h (Fig. 3; GLM: *Wald ӽ*_3_ *²* = 6.210, *P* = 0.10) and was similar across treatments after 24 h (*Wald ӽ*_3_ *²* = 1.462, *P* = 0.69).

### Survival in dependence of aggregation (experiment 2)

**Figure 4:**
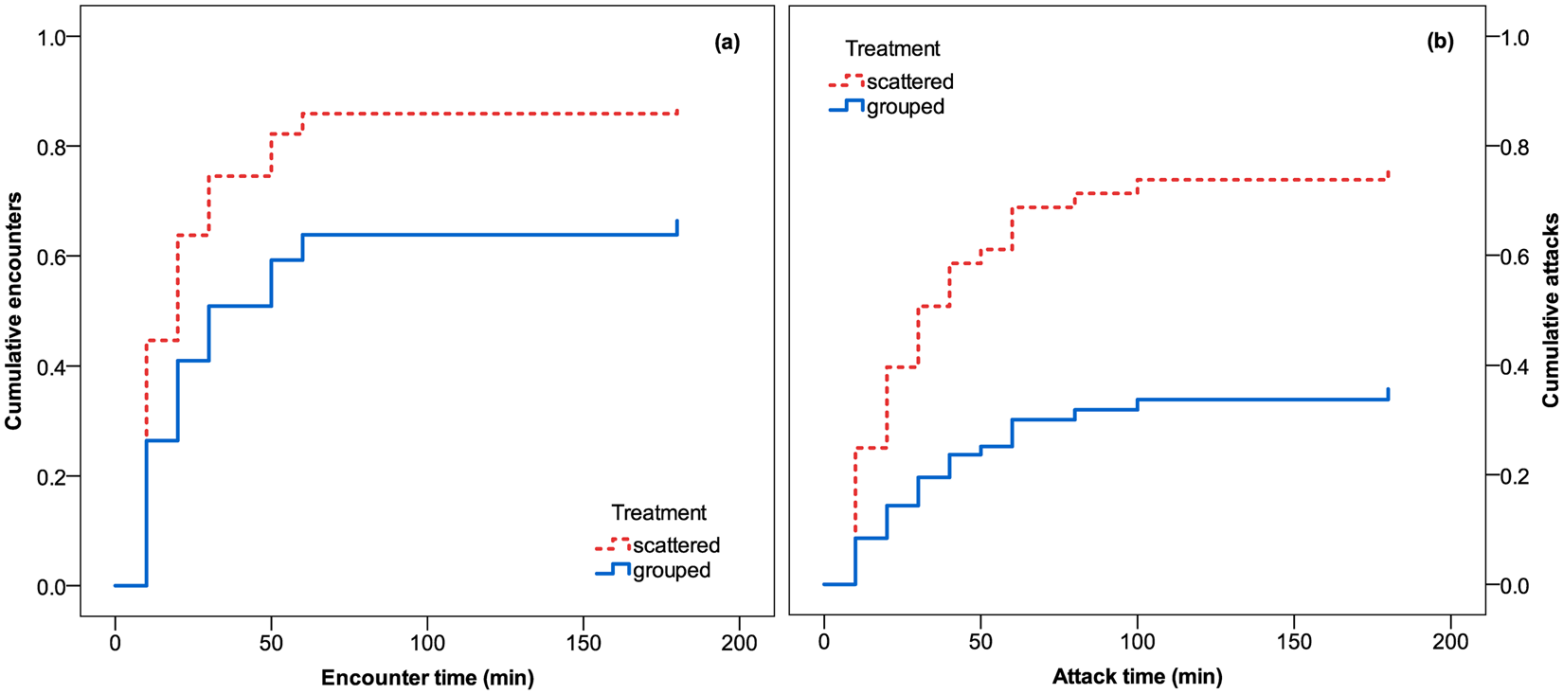
Prey aggregation-dependent risk of predator encounter and attack. Predator encounter (**a**) and attack (**b**) risk of four scattered or four grouped spider mite females on a leaf disc in presence of a predatory mite female, *Phytoseiulus persimilis* (Cox regression; *P* ≤ 0.05 for both encounter and attack). Encounters were scored when the predators touched a spider mite with the first pair of legs; attacks were scored when the predators successfully grasped a spider mite and started sucking it out.

Grouped spider mites were less likely and later encountered by the predatory mites than scattered spider mites (Fig. 4a; Cox regression: *Wald ӽ*_1_ *²* = 3.752, *P* = 0.05). Within 10 min after release, the predatory mites had encountered the first spider mite on 45% of leaf discs harboring four scattered individuals but only on 27% of discs harboring four grouped spider mites. Grouped spider mites were less likely and later attacked by the predatory mites than scattered spider mites (Fig. 4b; Cox regression: *Wald ӽ*_1_ *²* = 7.681, *P* = 0.006).

**Figure 5:**
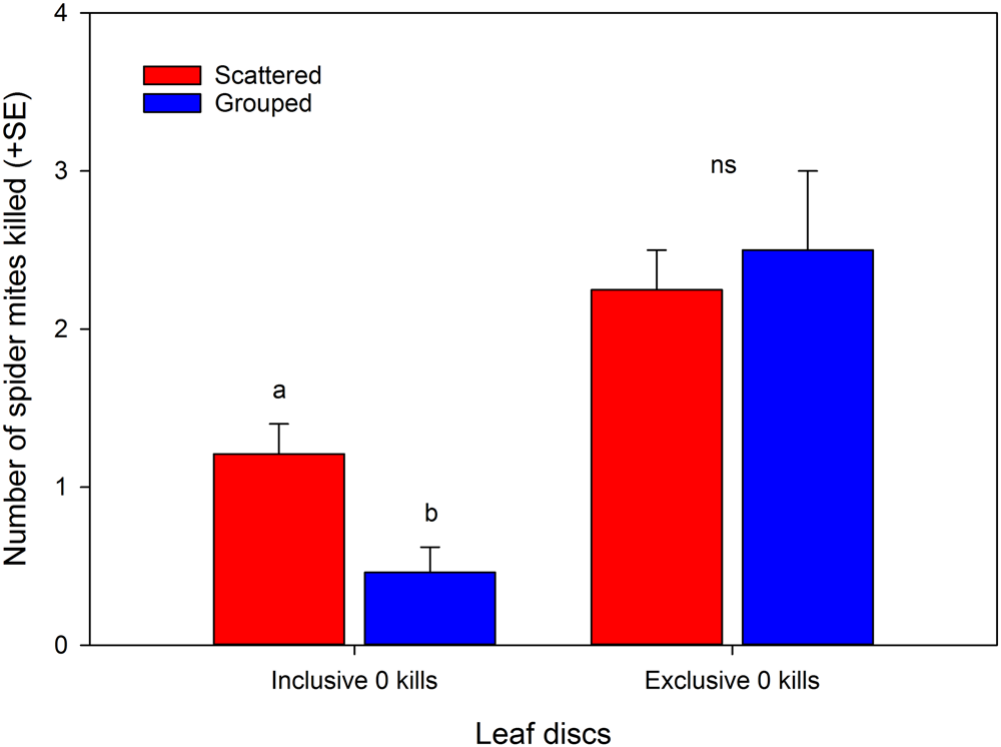
Mortality risk of grouped versus scattered prey. Number of scattered and grouped spider mite females, out of four per leaf disc, killed by a predatory mite female, *Phytoseiulus persimilis*, including and excluding discs without any killed spider mites (0 kills). Different letters above bars indicate significant differences between scattered and grouped spider mites (GLM; *P* < 0.01; ns for non-significant).

The predatory mites killed in total more scattered than grouped spider mites (Fig. 5; GLM: *Wald ӽ*_1_ *²* = 9.585; *P* = 0.002). Attack of one group member did not increase the risk of the other group members to be attacked, as compared to scattered spider mites: on leaf discs where at least one spider mite was killed, the total number of spider mites killed was the same for both treatments “scattered” and “grouped” (Fig. 5; GLM: *Wald ӽ*_1_ *²* = 0.138; *P* = 0.71).

## Discussion

Our study documents that adult females of two-spotted spider mites *T. urticae* form tighter aggregations when perceiving cues of the predatory mite *P. persimilis*. Graded predator cue availability and intensity did not modify the aggregative response of the spider mites, i.e. aggregation in the predator cue treatments differed from the no risk treatment but was similar across predator cue treatments. Our study provides evidence for the adaptive benefit of tighter aggregation. Grouped spider mites were less likely and later encountered and attacked by the predatory mites, hence had higher survival chances under predation risk, than scattered spider mites. While most previous pertinent studies were concerned with the encounter effect of prey aggregation on visually hunting predators^4, 16, 30, 56^, to the best of our knowledge, rigorous experiments on the effects of prey aggregation on prey survival and detection by predators orienting exclusively on chemosensory cues, like the predatory mites *P. persimilis*, have been lacking^4^.

The first experiment revealed that spider mite females aggregate more tightly in presence than absence of predatory mite cues, but the aggregation levels did not differ among treatments with predatory mite cues (representing low, medium, and high risk). Whether the spider mites are able to adjust the degree of aggregation in response to different species of predatory mites, posing low and high risks, remains to be tested. The spider mites were also more active in treatments with than without predatory mite cues, but the activity levels were similar across predatory mite cue treatments. Spider mites perceiving chemical traces of predatory mites^47^ commonly try to get away from areas perceived as potentially risky for themselves and/or their offspring, and thus show increased activity^25, 26^. Similar to our observations, Fernandez-Ferrari & Schausberger^26^ observed higher spider mite activity on leaves with than without predator cues but did not detect different activities on leaves with predatory mite traces with and without predator eggs. In general, higher activity, reflecting heightened stress levels caused by perception of cues indicating a risky environment, is an anti-predator response typically used by animals trying to escape from, or avoid, risky areas for their own safety and/or that of their offspring, and being devoid of possibilities to hide or access a refuge^1^. Whether prey increase or decrease their activity in response to predator cues may also depend on predator type, risk and hunting mode. For example, wood crickets increase their activity when perceiving cues of commonly encountered spiders, posing high predation risk, but decrease their activity in response to rarely encountered, and thus less risky, spiders^57^. Grasshoppers reduce their activity when threatened by sit-and-wait spider predators but increase their activity in response to highly mobile active spiders^58^.

Spider mites are inherently group-living^34, 37^ and benefit from Allee effects through the presence and webs of conspecifics^41^. The webs have also an important anti-predator function, with denser webbing providing better protection from predators^34^. Our study suggests that mere increase of aggregation, apart from, or additionally to, the webbing, is an efficacious anti-predator strategy of the spider mites. After 1 to 2 h, the inter-individual distances and the activity levels of the spider mites in the predatory mite cue treatments and the control started to converge, suggesting that (i) the predator traces gradually dissipated and became less repellent to the spider mites over time, and/or (ii) the spider mites adjusted their behavior by learning that the predator traces alone do not present a risk, because not followed by any consequences. Regarding (i), Le Goff *et al*.^59^ observed that aged spider mite webbing is less attractive to conspecifics than fresh webbing and suggested that the chemical cues on the webbing oxidized and/or degraded, or that volatile components vanished. Barnes *et al*.^60^ observed that wolf spiders responded more strongly to fresh than aged intraguild predator webs and excreta. Regarding (ii), Hackl & Schausberger^49^ observed higher activity in predator-naive than -experienced spider mites perceiving only traces, but not physical presence, of *P. persimilis*, suggesting learned predation risk management.

The number of spider mites present on the leaf discs did not differ among treatments. However, in all treatments, the number of spider mites present on the discs decreased after 24 h. This was possibly an artifact of the disproportionally long interval between the 3.5 and 24 h observation points, in which missing spider mites were not replaced or rescued anymore, and/or additionally and/or alternatively due to aging and spider mite damage, and associated decreased quality, of the leaves. With increasing leaf disc age and damage, more and more spider mite females may have started searching for new feeding sites, possibly followed by others^61^, and finally got stuck, and drowned, in the water surrounding the disc. The number of eggs was marginally lower and their aggregation was marginally higher in predatory mite cue treatments than the control after 3.5 h, but both parameters were similar across treatments after 24 h. Similar to our study, Grostal & Dicke^47^ observed that the number of eggs laid by spider mite females within 24 h did not differ between leaf discs with and without *P. persimilis* cues. Fernandez-Ferrari & Schausberger^26^ and Hackl & Schausberger^49^ observed that spider mite females lay their first egg later in presence than absence of *P. persimilis* cues, which could be due to energy trade-offs and/or egg retention^54^. We did not measure the exact timing of the onset of oviposition but the initial slight difference and later similarity in egg numbers was likely due to, at least partial, vanishing of the predatory mite cues, and/or the spider mites habituating to the cues^49^. Ovipositing spider mite females given a choice between sites with and without predatory mite cues avoid oviposition in the site with predatory mite cues^26, 47^. Thus, likely reasons for the marginally significant difference in egg aggregation after 3.5 h but lacking difference after 24 h, are the small size of the experimental discs and predatory mite cues covering the whole surface, leaving no safe areas. After 24 h, nearly all 16 virtual leaf sectors contained spider mite eggs.

The second experiment revealed that spider mite females clearly benefit from tighter aggregation. Scattered spider mites were significantly earlier encountered and attacked by *P. persimilis* than grouped spider mites. Proximately, disproportionally late detection of grouped spider mites, relative to scattered individuals, was due to the local prey searching strategy of *P. persimilis* orienting on tactile and volatile chemosensory cues^44, 62^. Similarly, Andersson *et al*.^22^ showed for moth that detectability of volatile infochemicals from the distance does not scale linearly to the number of cue emitters but proportional to its square root. Most strikingly, on leaf discs with at least one killed spider mite, the total number of killed spider mites was similar in both treatments “grouped” and “scattered”, indicating that detection of the group of four spider mites and attack of one group member did not increase the risk of the other three group members to be attacked, compared to four scattered spider mites. Possible initial benefits arising from attack abatement may later, after group detection and attack of the first group member, be opposed or even nullified if other group members are more likely killed than scattered prey individuals^4, 63^. In our experiment, these costs did not occur and may have been offset by the lack of webbing and associated chemical cues, which are important for locally guiding *P. persimilis* to individual prey items^43, 45^.

Johannesen *et al*.^63^ examined the effects of chironomid larvae aggregations on detection and killing by three-spined sticklebacks, which primarily use visual but also chemosensory cues and lateral line detection to locate prey. Johannesen *et al*.^63^ manipulated water turbidity to decrease the importance of visual and mechanosensory cues and increase the importance of chemosensory cues. They observed that dispersed prey were significantly earlier detected and consumed than semi-dispersed and aggregated prey. This result is similar to our finding of aggregated spider mites being later encountered and attacked than scattered spider mites. However, different from our experiments, Johannesen *et al*.^63^ observed that, after prey group detection by the sticklebacks, group members were more likely attacked and consumed than dispersed prey individuals. Contrary, in our experiments, after prey group detection and attack of the first prey individual, the other group members were not at higher risk to be killed than scattered individuals. The difference between our findings and those of Johannesen *et al*.^63^ is likely based on differences in the used sensory modalities and searching strategies of the sticklebacks and predatory mites. After group detection, in the sticklebacks, visual cues or a combination of visual, mechanosensory and chemosensory cues came into play to locate other group members. This was not the case in *P. persimilis*, which exclusively use chemosensory cues to detect and recognize prey. Moreover, under natural settings, where the mites are not fixed to the surface, as in our experiment, but movable, the predator:prey body size ratio, and resulting handling time of the predator, influences the escaping chances of other group members following attack of the first individual. Regarding the anti-predator benefits of group-living, predator satiation effects not only depend on group size^64^ but also on predator-prey body size ratios^65^. At the same group size, individuals within groups composed of large prey will benefit more from predator satiation effects than those within groups of small prey. A gravid *P. persimilis* female needs several minutes to attack and suck out an adult spider mite female, which has approximately the same size as its predator, allowing nearby spider mite females to escape. Time for escaping scales with prey size-dependent functional response and predator satiation, i.e. predator satiation lasts longer after ingesting a large than small prey individual^65, 66^.

## Conclusions

Two-spotted spider mites *T. urticae* aggregate more tightly when perceiving cues of the predatory mite *P. persimilis*. Tighter aggregation is adaptive because increasing the survival chances of the spider mites under predation risk. Proximately, the benefits of tighter aggregation arise from the encounter effect and the dilution effect, which combination is dubbed attack abatement^11^. Possible trade-offs opposing the dilution component of attack abatement, such as more likely encounters and attacks on other group members after detection of the first group member, relative to solitary prey individuals, did not occur.
